# Invasive Mucinous Adenocarcinoma Presenting as Cavitary Lung Disease: A Case Report and Narrative Review

**DOI:** 10.7759/cureus.102395

**Published:** 2026-01-27

**Authors:** Anna Haymov, Mohammed Alsaggaf, Japleen Kaur

**Affiliations:** 1 Department of Neurology and Neurosurgery, Lake Erie College of Osteopathic Medicine, Elmira, USA; 2 Department of Pulmonology, Critical Care and Sleep Medicine, Olean Medical Group, Olean, USA; 3 Department of Medicine, Government Medical College, Amritsar, Amritsar, IND

**Keywords:** adenocarcinoma lung, ca lung, cavitary lung disease, invasive mucinous adenocarcinoma, lung cancer, thoracic radiology

## Abstract

Invasive mucinous adenocarcinoma (IMA) is a rare subtype of lung adenocarcinoma that often presents with non-specific or misleading radiographic findings. We report a case of a 71-year-old female patient, non-smoker, who presented with progressive dyspnea on exertion and chronic cough unresponsive to multiple antibiotic regimens. Imaging revealed bilateral cavitary lung lesions. Comprehensive infectious and autoimmune evaluations, including bronchoscopy with bronchoalveolar lavage, were negative, and transbronchial biopsy was non-diagnostic. A surgical lung biopsy ultimately confirmed IMA. This case underscores the diagnostic challenges posed by cavitary IMA, which may resemble benign infectious or inflammatory processes, particularly in patients without conventional risk factors, leading to delayed diagnosis and treatment.

## Introduction

Mucinous adenocarcinoma of the lung is a rare histologic subtype of lung adenocarcinoma, accounting for approximately 2-5% of all primary pulmonary adenocarcinomas [1,2]. Formerly classified as mucinous bronchioloalveolar carcinoma under the 2004 WHO system, it has since been redefined in the 2015 WHO classification as invasive mucinous adenocarcinoma (IMA) due to its distinct molecular, radiographic, and pathological characteristics [3].

IMA is characterized by tall columnar epithelial cells with abundant mucin production and often demonstrates a lepidic pattern of growth with invasive components [4]. Clinically, it may present as a solitary nodule or diffuse bilateral involvement, manifesting with cough, dyspnea, or recurrent pneumonias [5]. While solid tumors are more common, cavitary presentations, such as in this case, are uncommon and frequently misdiagnosed as infectious or inflammatory processes, particularly when located in the lower lobes [6,7].

Radiographically, IMAs can mimic organizing pneumonia or present as multifocal consolidations and nodular opacities. Cavitary lesions may result from valve mechanism changes or mucin-filled spaces and are typically peripherally distributed [6,8]. Prognosis is generally poorer than that of non-mucinous adenocarcinoma, especially in multifocal disease, which often demonstrates resistance to conventional chemotherapy or targeted agents [9,10].

This case highlights the diagnostic challenge posed by cavitary pulmonary lesions and emphasizes the importance of considering mucinous adenocarcinoma in the differential diagnosis, particularly in patients without infectious risk factors and when initial antibiotic therapies fail.

## Case presentation

**Figure 1:**
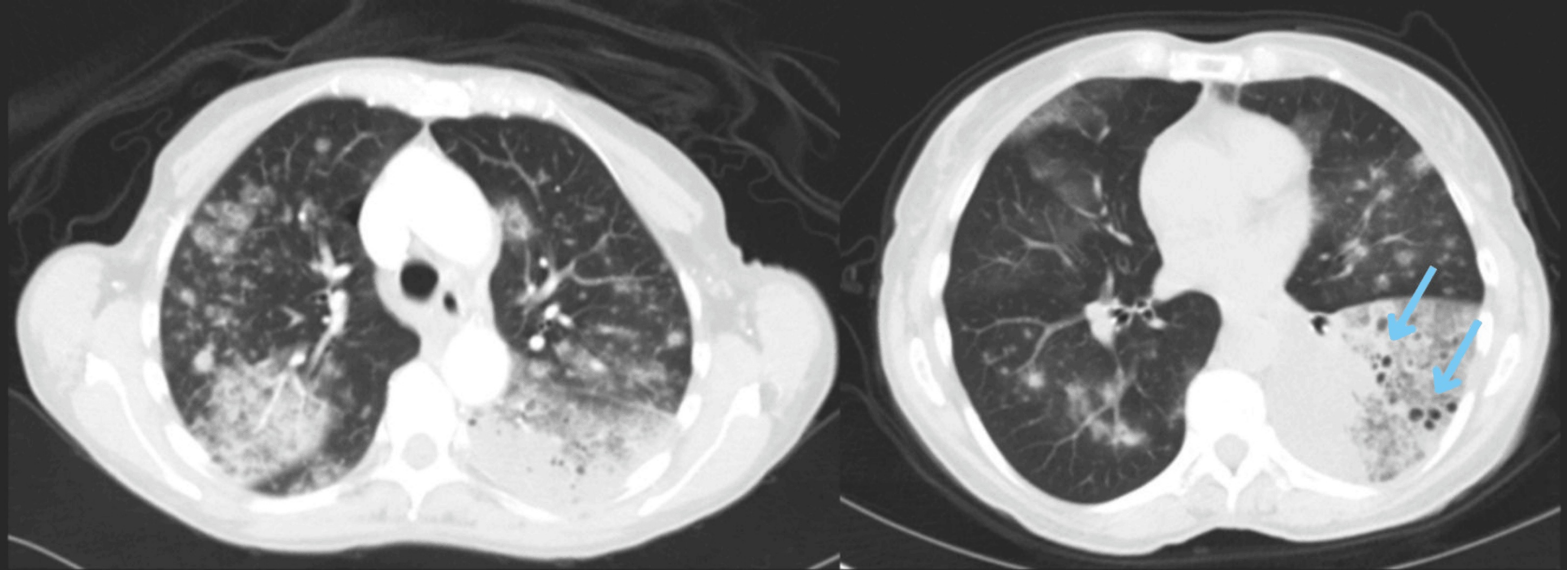
Chest CT without contrast showing extensive infiltrates bilaterally, mostly at the left lung base with some cavities (blue arrows).

A 71-year-old female patient, non-smoker, presented to the outpatient pulmonology clinic with a two-month history of progressive cough and exertional dyspnea. Initial chest X-ray (CXR) demonstrated bilateral opacities consistent with pneumonia, for which she was treated with oral amoxicillin-clavulanate, azithromycin, and intranasal fluticasone, with no symptomatic relief. A follow-up CXR revealed worsening bilateral infiltrates, prompting a short course of oral corticosteroids and levofloxacin, again without clinical improvement. A chest CT performed a month later demonstrated worsening bilateral airspace disease with new cavitary lesions, raising suspicion for atypical infection or septic emboli (Figure 1).

The patient presented to the emergency department 10 days later for worsening hypoxia (oxygen saturation in 90s) and fever (101°F) and was admitted to the hospital. Comprehensive labs, including complete blood count (CBC) and coagulation panel, were completed (Table 1), mainly showing non-specific neutrophilia and elevated WBC count, suggesting an infectious etiology. Platelet count and prothrombin time were also slightly elevated (Table 1).

**Table 1:** Complete blood count and coagulation panel on admission

	Patient’s Values	Reference Values
White blood cells	13.2 K/mm^3^	4-10.5 K/mm^3^
Red blood cells	4.18 million/uL	4.2-5.4 million/uL
Hemoglobin	12.6 g/dL	12.5-16.0 g/dL
Hematocrit	37.7%	37-47%
Platelets	478 K/mm^3^	150-450 K/mm^3^
Neutrophil %	78.4%	40-74%
Lymphocyte %	7.8%	19-48%
Monocyte %	12.8%	3-9%
Eosinophil %	0.5%	0-7%
Basophil %	0.5%	0-2%
Erythrocyte sedimentation rate	26 mm/hour	0-33 mm/hour
Prothrombin time	14.6 seconds	9.7-13.7 seconds
Partial thromboplastin time	30.9 seconds	27.9-39.9 seconds
International normalized ratio	1.22	0.77-1.16

**Figure 2:**
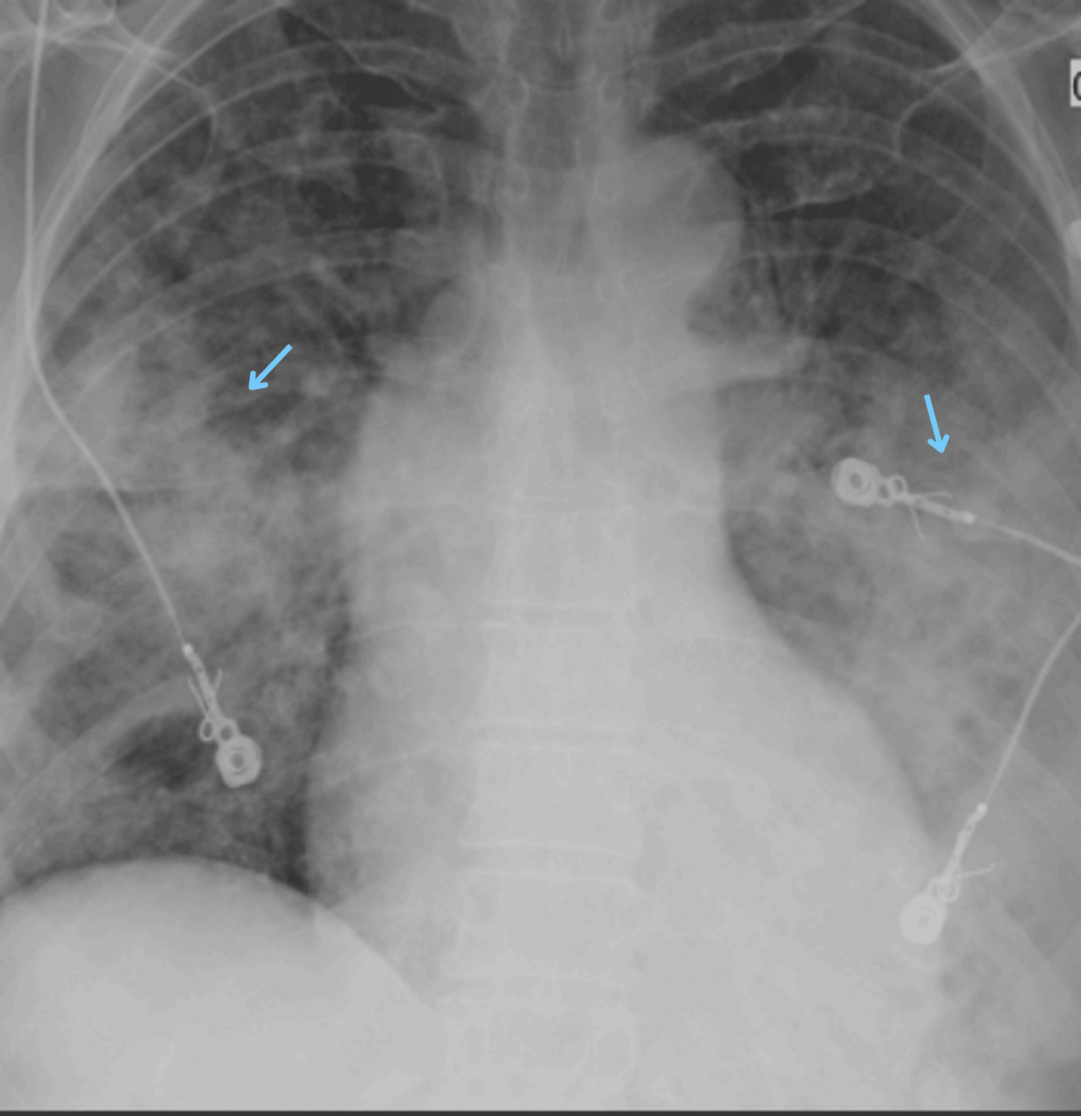
Chest X-ray completed on admission to the hospital, showing diffuse infiltrates mainly in the lower lobes and more prominent on the left (blue arrows).

The comprehensive metabolic panel (CMP) was within normal limits. CXR showed diffuse infiltrates that are worse on the left than the right (Figure 2).

**Figure 3:**
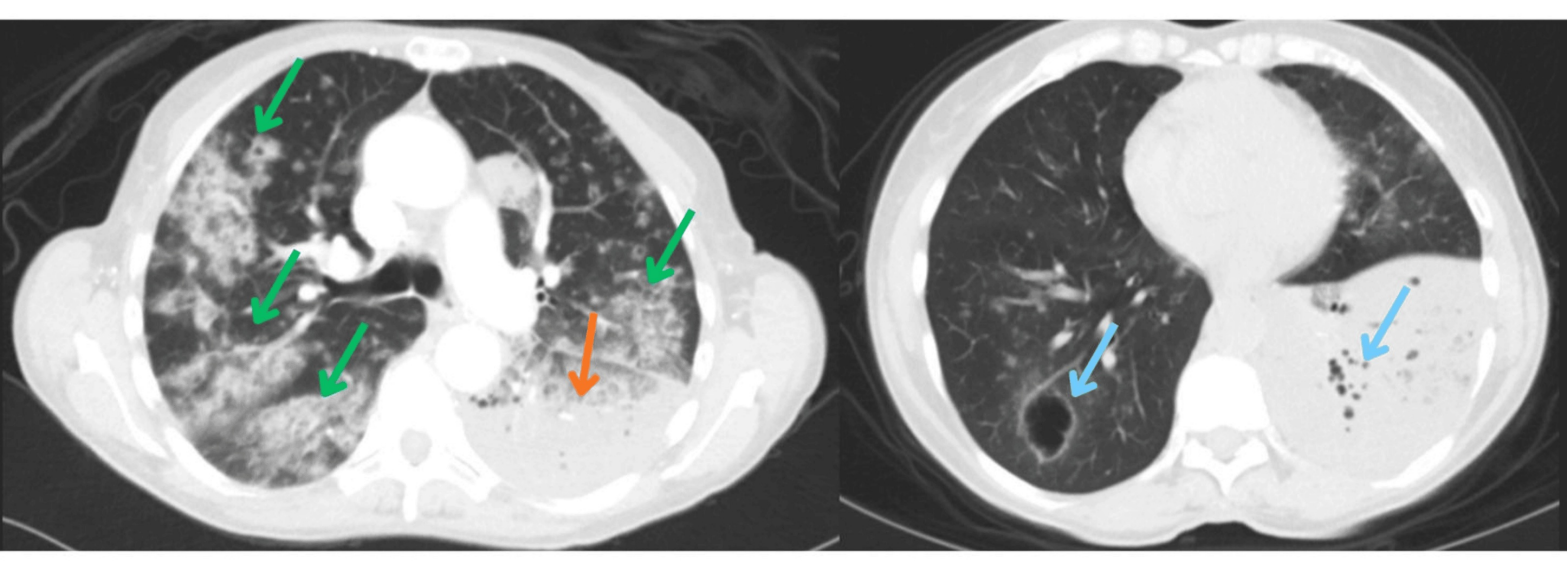
Computed tomography angiography with contrast showing severe multifocal infiltrates (green arrows) with diffuse bilateral cavitating pulmonary lesions (blue arrows). There is a near-complete collapse of the inferior segment of the left lower lobe (orange arrow).

Empiric intravenous vancomycin, piperacillin-tazobactam, and azithromycin were initiated. CT angiography (CTA) ruled out pulmonary embolism but revealed bilateral cavitating lesions and near-complete collapse of the inferior left lower lobe that had progressed since the first CT (Figure 3).

Blood and sputum cultures, as well as infectious serologies, including testing for tuberculosis, fungal pathogens (Cryptococcus, Coccidioides, Blastomyces, Histoplasma, Aspergillus), MRSA, HIV, and COVID-19, were negative.

Bronchoscopy with bronchoalveolar lavage (BAL) and transbronchial biopsy (TBB) was performed on the third day of her hospitalization, targeting the left lower lobe. Pathology revealed reactive alveolar epithelial proliferation and interstitial inflammation, suggestive of chronic inflammatory response versus bronchioloalveolar carcinoma. BAL cell count was neutrophil-predominant (84%), with no evidence of malignant cells or infectious organisms. However, BAL alone is non-diagnostic and requires further testing and evaluation. The pathology report did not find evidence of malignancy, and the specimen was sent to an external lab for re-evaluation, which also returned negative. BAL cultures were negative for bacteria, Coccidioides, and Blastomyces. As infectious workup continued to provide negative results, an autoimmune workup was performed to rule out other etiologies, including ANCA, ANA, RF, PR3, myeloperoxidase antibodies, dsDNA, SSA/SSB, and Scl-70. However, all these tests were negative.

Despite seven days of IV antibiotics, there was no clinical or radiographic improvement. A transesophageal echocardiography ruled out infective endocarditis. A positron emission tomography (PET) scan performed on February 14 showed bilateral consolidations, nodules with ground-glass halo sign, and moderate FDG uptake. She was discharged home stable on room air with persistent symptoms and referred for surgical lung biopsy. Two months after her initial presentation, the patient returned after undergoing a video-assisted thoracoscopic biopsy at an outside facility, which confirmed mucinous lung adenocarcinoma. Given her rapid decline and disease progression, she elected for comfort care and passed away two days later.

## Discussion

Literature review

Cavitary lung lesions are classically attributed to infectious, autoimmune, or necrotic squamous cell carcinomas; however, an increasing number of reports describe IMA presenting with cavitation, a form that can closely mimic benign or infectious processes such as abscesses, cysts, or emphysema. These atypical presentations contribute to diagnostic delays, limiting the time available for therapeutic intervention and shifting clinical effort toward establishing the diagnosis rather than optimizing oncologic management and outcomes [11-13].

A review of reported cases of lung adenocarcinoma presenting with lung cavities (Table 2) reveals that the subtype cavitary IMA occurs across a wide age range (37-82 years) and in both sexes, though females slightly predominate. Smoking history is variable; many patients, like ours, are non-smokers, emphasizing the controversial role of smoking in disease occurrence and progression. Radiographically, CT findings frequently include thin- or thick-walled cavities, ground-glass opacities, and bilateral or multifocal involvement, often in the lower lobes [11-22]. Some cases describe distinctive imaging signs, such as the “tambourine sign” [15], or irregular air-filled cavities with centrilobular nodules [11]. These patterns commonly lead to initial misdiagnoses of infectious or autoimmune etiologies.

**Table 2:** Comparative summary of cavitary invasive mucinous adenocarcinoma cases reported in the literature. *CT findings other than cavities CT, computed tomography; MAC, Mycobacterium avium complex; PPY, packs per year

	Our Case	Carreto et al. [11]	Verma et al. [15]	Castillo et al. [17]	Nakamura et al. [18]	Fujita et al., Case 1 [19]	Fujita et al., Case 2 [19]	Yeon et al. [20]	Sakai et al. [21]	Araiza et al. [22]
Age	71	62	37	40	78	82	81	63	72	69
Sex	Female	Female	Female	Female	Female	Male	Female	Male	Male	Male
Smoking history (PPY)	None	30	None	Unknown	None	Unknown	Unknown	15	52.5	22.5
CT findings*	Ground-glass opacities	Centrilobular nodules	“Tambourine sign”	-	Nodules	Fungus ball with interstitial pneumonia	Nodules	Nodules	Nodules	Nodules
Cavities	Yes	Yes	Yes	Yes	Yes	Yes	Yes	Yes	Yes	Yes
Final diagnosis	Mucinous adenocarcinoma	Mucinous adenocarcinoma	Mucinous adenocarcinoma	Mucinous adenocarcinoma	Pulmonary lymphangitis carcinomatosis due to pulmonary invasive mucinous adenocarcinoma	Pulmonary aspergillosis and lung adenocarcinoma	MAC lung disease and lung adenocarcinoma	Well-differentiated adenocarcinoma with lepidic growth	Adenocarcinoma with solid, papillary, and lepidic subtypes	Poorly differentiated adenocarcinoma
Diagnostic method	Surgical biopsy	Surgical biopsy	Chest CT and ultrasound-guided biopsy	Ultrasound-guided transbronchial biopsy	Autopsy	Bronchoscopy with biopsy	Surgical biopsy	Surgical biopsy	Surgical biopsy	Ultrasound-guided transbronchial biopsy
Time passed to final diagnosis (months)	3	5	72	6	36	Unknown	Unknown	10	Unknown	2
Treatment	Comfort care	Chemotherapy	Chemotherapy	Unknown	Antibiotics and palliative care	Antifungals	Lobectomy and combination therapy for MAC lung disease	Lobectomy	Lobectomy and wedge resection	Stereotactic radiosurgery and chemotherapy

Diagnostic confirmation has typically required surgical lung biopsy, as less invasive methods such as transbronchial or percutaneous biopsy often yield non-specific inflammatory changes. The time from symptom onset to definitive diagnosis ranged widely in prior cases (from two months to six years), highlighting the diagnostic difficulty associated with cavitary IMA.

Treatment approaches also vary by disease stage and patient factors. Some patients underwent surgical resection (lobectomy or wedge resection), while others received systemic chemotherapy, palliative care, or, in cases of coexisting infection such as MAC or aspergillosis [18-19], antimicrobial therapy. Prognosis remains guarded, particularly for multifocal or advanced cases, and elderly patients are often excluded from aggressive interventions.

Case discussion

Our patient, a 71-year-old non-smoking female with bilateral cavitary lesions unresponsive to antibiotics, exemplifies this uncommon and diagnostically challenging presentation. Her clinical course closely mirrors previously described cases in which IMA mimicked chronic infection, leading to multiple rounds of antimicrobial therapy before definitive diagnosis.

While adenocarcinomas with cavitation are more frequently reported in older males with smoking histories, elevated carcinoembryonic antigen (CEA), or bulky peripheral tumors [13,14], our case underscores that IMA may occur outside of these typical demographics.

The pathophysiologic mechanisms of cavitation in adenocarcinoma include mucin overproduction, tumor necrosis, and bronchial obstruction creating a check-valve effect [15,16]. Radiographically, malignant features such as asymmetric wall thickening, mural nodules, internal septations, or adjacent ground-glass opacities should prompt suspicion for malignancy even when cavities appear thin-walled [12,15]. These findings were evident in our case and, together with progressive imaging changes, warranted biopsy confirmation.

IMA frequently presents with multifocal cavitary nodules or consolidations that mimic organizing pneumonia or necrotizing infection [17,18]. In our patient, this overlap delayed diagnosis, as multiple infectious and autoimmune evaluations were negative. Her bilateral distribution, air-fluid levels, and segmental collapse were consistent with previously described IMA cases.

AAlthough adenocarcinomas with lepidic or adenocarcinoma in situ/microinvasive adenocarcinoma patterns are less likely to cavitate, papillary-predominant and invasive subtypes have been associated with cavitation and poorer outcomes [13]. The rapid progression and extensive disease in our case align with these aggressive variants.

Importantly, IMA may coexist with infectious processes such as *Mycobacterium avium* complex or aspergillosis, further obscuring the malignancy [19]. While cultures were negative in our case, clinicians must remain alert to the potential for dual pathology.

As emphasized in prior reports, non-resolving cavitary lesions, especially those with growth, consolidation, or internal soft-tissue elements, should prompt early biopsy, even if PET findings are equivocal [20-22]. In retrospect, earlier tissue diagnosis might have led to more timely recognition of malignancy.

This case reinforces the importance of maintaining a high index of suspicion for malignancy in patients with cavitary lung lesions unresponsive to antimicrobial therapy, even in the absence of traditional risk factors. Prompt consideration of mucinous adenocarcinoma in the differential diagnosis may allow for earlier intervention and, possibly, improved outcomes.

## Conclusions

Pulmonary cavitary IMA poses a significant diagnostic challenge because it can closely mimic benign and infectious lung diseases. This case underscores the importance of maintaining a broad differential diagnosis when evaluating non-resolving cavitary lesions, particularly in patients without traditional risk factors such as smoking. Exclusive reliance on imaging findings and empiric antimicrobial therapy may delay definitive diagnosis, narrowing the window for timely implementation of targeted treatment strategies. While no definitive treatment has been proven to improve survival, early and accurate diagnosis provides valuable time to explore additional therapeutic options and pursue strategies that may ultimately lead to remission or a cure.
